# Treatment of Disease by Electricity

**Published:** 1890-02-15

**Authors:** 


### TREATMENT OF DISEASE BY ELECTRICITY.

In a former retrospect we noticed the treatment of cancer, as reported by electricity.

At a recent meeting of the Harveian Society, Dr. Cagney read a paper on the administration of certain drugs by electricity, stating that some drugs can be made to pass through the animal tissues between the poles of a galvanic current, but the treatment is best adapted to diseases of the skin, of the mucus membranes, or of tumours immediately beneath. Nodes and gummata in accessible positions seem to yield readily to iodide of potassium thus introduced. The advantages claimed are that it is possible to administer a drug, not otherwise tolerated in every constitution, in a manner least liable to disturb the system; that it is conveyed directly to the part, which at the same time is benefited by the action of the galvanic current. Great current strength is not required. The electrodes may be either sponges holding a solution of iodide of potassium, or a conducting tube filled with the fluid. We are not, however, sanguine that this method of treating disease will become established, although it is possible. Iodide of potassium is often employed in large doses before it has any effect on syphilitic deposits, and it may be questioned whether sufficiently large quantities can be conveyed into the system by electricity. Moreover, syphilis is a constitutional malady, and will not be cured by attacking any local lesion it may cause. At the meeting of the Harveian Society above referred to, Dr. Buzzard observed that it was evident a very strong current must be used to cause a drug to pass through the tissues to any considerable depth; and Dr. Cagney confessed that only slight results could be expected when the current had to pass through the cuticle. Moreover, the questions occur to us whether or not any drug really does pass with the electrical current, and if so passing, whether any is deposited in the tissues.

